# Effectiveness and cost-effectiveness of combination therapy versus monotherapy in malignant melanoma

**DOI:** 10.1186/s40545-023-00611-7

**Published:** 2023-09-25

**Authors:** Dilay Özdemir, Melanie Büssgen

**Affiliations:** 1 University of Hamburg, Hamburg, Germany; 2 Hamburg Center for Health Economics, University of Hamburg, Hamburg, Germany

**Keywords:** Cost-effectiveness, Effectiveness, Melanoma, Malignant melanoma, Oncology, Immune checkpoint inhibitors, BRAF inhibitor, MEK inhibitor, Targeted therapy, Combination therapy

## Abstract

**Background:**

Until 2010, stage III or IV malignant melanoma (MM) had a poor prognosis. The discovery of immune checkpoint inhibitors (ICIs) in 2011 changed the treatment landscape. Promising results in patient survival with a checkpoint inhibitor prompted research into combination therapies. In 2016, the first combination therapy has been approved as first-line therapy for advanced MM.

**Objective:**

The aim of this work is to investigate to what extent combination therapy is (cost-)effective compared to monotherapy in stage III or IV MM.

**Methods:**

A systematic literature search was performed (Web of Science, PubMed, PubPharm, EconLit, and Cochrane Library); searching for publications published over the past decade that examine the cost-effectiveness in terms of cost/QALY and the effectiveness in terms of survival and response of combination therapy in comparison to monotherapy in stage III or IV MM patients.

**Results:**

A total of 11 randomized controlled trials (RCTs) and five cost–utility analyses met our inclusion criteria. Nine clinical trials demonstrated superiority of combination therapy over monotherapy. The combination of B-rapidly accelerated fibrosarcoma (BRAF) protein and mitogen-activated kinase (MEK) protein inhibitors is not cost-effective in any country. Three analyses demonstrate the cost-effectiveness of combination therapy with ICI compared to monotherapy.

**Conclusion:**

Combination therapy is more effective compared to monotherapy. While combined ICIs are cost-effective compared to monotherapy, this is not the case for the combination of BRAF and MEK inhibitors.

## Introduction

Skin cancer is the 17th most common cancer worldwide [1] with an incidence of approximately 325,000 [2]. Malignant melanoma—which is a type of skin cancer that develops from the pigment-producing cells known as melanocytes—is among the most aggressive skin cancers and causes more than 90% of all skin cancer deaths in Germany [3]. The incidence is increasing yearly across the world [4]. Whereas early-stage MM is curable, metastatic melanomas are difficult to treat. The 5-year survival rate for a MM that has spread to nearby lymph nodes is 62% [5, 6] and for MM that has spread to distant lymph nodes or other areas of the body ranges from 10 to 25% [7].

Adjuvant treatment of stage III or IV MM was limited to chemotherapy until 2010. The discovery of ICIs revolutionized the therapeutic landscape. The introduction of ipilimumab (IPI) (CTLA-4-inhibitor), the first checkpoint inhibitor approved by the FDA in 2011, demonstrated an increase in overall survival (OS) compared to chemotherapy. While the OS rate at 24 months with IPI was 23.5%, it was 13.7% with chemotherapy [8]. This breakthrough innovation was also approved in Europe in 2011 [9]. The discovery of the immune checkpoint PD-1 as another therapy-relevant target has led to the development of several PD-1 antibodies. In June 2015, the first anti-PD-1 antibody nivolumab (NIVO) was approved in Europe [10]. In the same year, the European Medicines Agency (EMA) granted approval for the second PD-1 antibody pembrolizumab (PEM) [9].

In recent years, the number of combination therapies available on the drug market has risen rapidly. Combination therapies are drugs that contain more than one active ingredient. The fixed combination of two substances in one drug is intended to simplify administration for patients and lead to an increase in adherence to therapy. Compared with monotherapy, this is expected to result in better efficacy and thus lower costs. A combination therapy of NIVO plus IPI, resulted for the first time in improved effectiveness compared to monotherapy with IPI [11]. The study results led to its approval as first-line therapy in patients with advanced melanoma in Europe in 2016 [12]. Furthermore, the discovery of the BRAF mutation in melanoma led to the approval of the BRAF inhibitors vemurafenib (VEM) in 2011 and dabrafenib (DAB) in 2013, after which the MEK inhibitors cobimetinib (COB) and trametinib (TRAM) were approved for combination therapy with BRAF inhibitors [7]. DAB plus TRAM showed an improvement in OS compared to DAB and VEM [13, 14]. Thus, DAB plus TRAM, the first targeted combination therapy for adults with advanced melanoma with BRAF mutation, was approved in Europe in 2015 [15]. To date, the cost-effectiveness of these combination therapies compared to monotherapy remains open.

However, the long-term potential of combination therapy remains controversial: For example, the fixed combinations limit individual adjustment of the dose regimen, which can lead to a reduction in efficacy. In addition, current knowledge about the safety of combination drugs is not comprehensive. Accordingly, the long-term effect of combination drugs compared to monotherapy on the health care systems remains controversial. Despite the approval of combination therapies, clinical data are not yet fully mature to assess long-term effectiveness. In addition, combination therapy is associated with additional costs. Given the attention devoted to pharmaceutical costs being at an all-time high, the issue of cost-effectiveness of these innovative combination therapies is of great importance for reimbursement decisions. Thus, the aim of this paper is (1) to give an overview of conducted MM clinical trials in the past decade and (2) to evaluate the effectiveness and cost-effectiveness of combination therapy compared to monotherapy as first-line treatment of stage III or IV melanoma based on the available evidence. Result parameters include therapeutic-clinical as well as economic parameters.

## Methodology

**Fig. 1:**
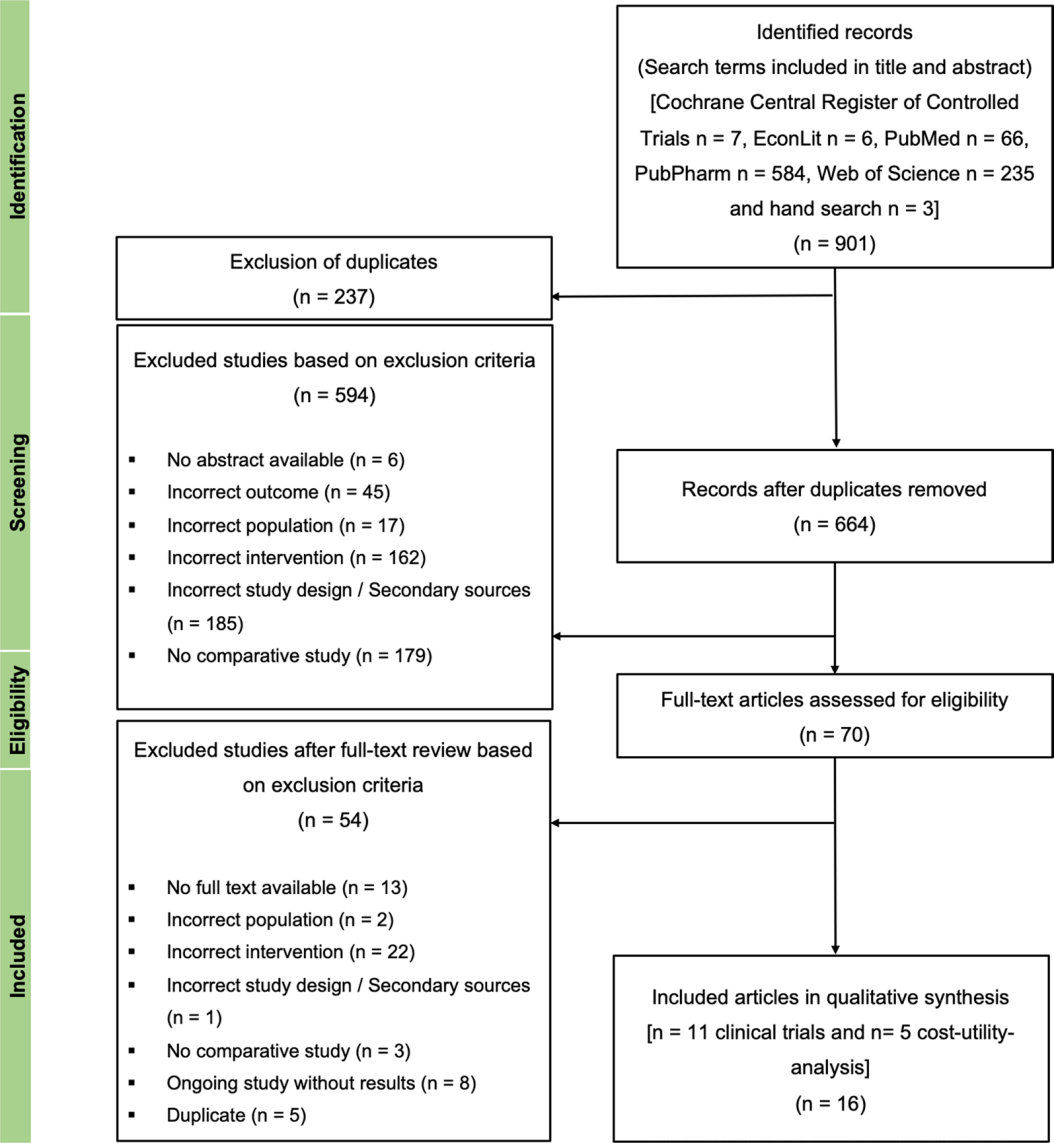
PRISMA flowchart

Systematic literature searches [16] for primary literature were conducted in the Web of Science, PubMed, PubPharm, EconLit, and Cochrane databases. The first search took place on February 12, 2022 and the last on June 11, 2022. In addition, a hand search of the publication reference lists was conducted. The PRISMA flowchart diagram is shown in Fig. 1.

## Results

### Study characteristics of the clinical trials

All investigated RCTs were multicenter phase III studies that evaluated treatment-naïve stage III or IV patients. Eight studies are labeled as double-blind and three as open-label. The RCTs were published between 2015 and 2021 and were sponsored by the pharmaceutical industry. All studies included data on a homogeneous patient population varying from 423 to 945 patients aged 18 years or older. The average age of patients is over 55 years. The proportion of women is lower than the proportion of men (40:60). Six studies included only patients with BRAF mutation and one study only patients with BRAF wild-type. Four studies included both BRAF mutation and BRAF wild-type patients. An overview of the 11 included RCTs is presented in Table 1.

**Table 1:** Overview of included randomized controlled trials

			Sample							PFS [%]							OS [%]									
Authors, year, countries [study name]	Intervention	Comparator	*N *	Randomization	Average age	Women [%]	Blinding	Mean observation time [months]	Median PFS [months]	12	24	36	48	60	78	Median OS [months]	12	24	36	48	60	78	OR [%]	TRAE of grade 3 or 4 [%]	Sponsor	Conclusion
*PD-1-Inhibitor plus CTLA-4-Inhibitor versus CTLA-4-Inhibitor or PD-1-Inhibitor*																										
Hodi et al. 2018, A, AU, B, CDN, CH, CS, D, DK, E, F, FIN, GB, I, IR, ISR, NO, NL, NZ, PL, S, US [CheckMate 067]	[1] NIVO (1 mg/kg) + IPI (3 mg/kg) once every three weeks for 4 doses, then NIVO (3 mg/kg) only every 2 weeks	[2] IPI (3 mg/kg) once every 3 weeks for a total of 4 doses + placebo [3] NIVO (3 mg/kg) once every 2 weeks + placebo	*N*= 945 [1] 314 [2] 315 [3] 316	1:1:1	59.6	35.4	DB	[1] 46.9 [2] 18.6 [3] 36.0	[1] 11.5 [2] 2.9 [3] 6.9 [1] vs. [2]: HR: 0.42 [95% CI: 0.35–0.51; *p*<0.0001] [1] vs. [3]: HR: 0.79 [95% CI: 0.64–0.96]*	n.s.	n.s.	n.s.	[1] 37 [2] 9 [3] 31	n.s.	n.s.	[1] >48.0 [2] 19.9 [3] 36.9 [1] vs. [2]: HR: 0.54 [95% CI: 0.44–0.67; *p*<0.0001] [1] vs. [3]: HR: 0.84 [95% CI: 0.67–1.05]*	n.s.	n.s.	n.s.	[1] 53 [2] 30 [3] 46	n.s.	n.s.	[1] 58 [2] 19 [3] 45	[1] 59 [2] 28 [3] 22	Bristol-Myers Squibb	NIVO plus IPI is more effective than IPI and NIVO in stage III or IV patients.
Larkin et al. 2019a, A, AU, B, CDN, CH, CS, D, DK, E, F, FIN, GB, I, IR, ISR, NO, NL, NZ, PL, S, US [CheckMate 067]	[1] NIVO + IPI	[2] IPI + placebo [3] NIVO + placebo	*N*= 945 [1] 314 [2] 315 [3] 316	1:1:1	59.6	35.4	O	[1] 54.6 [2] 18.6 [3] 36.0	[1] 11.5 [2] 2.9 [3] 6.9 [1] vs. [2]: HR: 0.42 [95% CI: 0.35–0.51; *p*<0.001] [1] vs. [3]: HR: 0.79 [95% CI: 0.64–0.96]*	n.s.	n.s.	n.s.	n.s.	[1] 36 [2] 8 [3] 29	n.s.	[1] >60 [2] 19.9 [3] 36.9 [1] vs. [2]: HR: 0.52 [95% CI: 0.42–0.64; *p*<0.001] [1] vs. [3]: HR: 0.83 [95% CI: 0.67–1.03]*	n.s.	n.s.	n.s.	n.s.	[1] 52 [2] 26 [3] 44	n.s.	[1] 58 [2] 19 [3] 45	[1] 59 [2] 28 [3] 23	Bristol-Myers Squibb	The 5-year analysis continues to show an improvement in effectiveness with NIVO plus IPI compared with IPI and NIVO.
Wolchok et al. 2021a, A, AU, B, CDN, CH, CS, D, DK, E, F, FIN, GB, I, IR, ISR, NO, NL, NZ, PL, S, US [CheckMate 067]	[1] NIVO + IPI	[2] IPI + placebo [3] NIVO + placebo	*N*= 945 [1] 314 [2] 315 [3] 316	1:1:1	59.6	35.4	O	[1] 57.5 [2] 18.6 [3] 36.0	[1] 11.5 [2] 2.9 [3] 6.9 [1] vs. [2]: HR: 0.42 [95% CI: 0.35–0.51; *p*<0.0001][1] vs. [3]: HR: 0.79 [95% CI: 0.65–0.97]*	n.s.	n.s.	n.s.	n.s.	[1] 36 [2] 8 [3] 29	[1] 34 [2] 7 [3] 29	[1] 72.1 [2] 19.9 [3] 36.9[1] vs. [2]: HR: 0.52 [95% CI: 0.43–0.64; *p*<0.0001] [1] vs. [3]: HR: 0.84 [95% CI: 0.67–1.04]*	n.s.	n.s.	n.s.	n.s.	[1] 52 [2] 26 [3] 44	[1] 49 [2] 23 [3] 42	[1] 58 [2] 19 [3] 45	[1] 59 [2] 28 [3] 24	Bristol-Myers Squibb	The 6.5-year analysis continues to demonstrate an improvement in effectiveness with the combination drug over IPI and NIVO
*BRAF-inhibitor plus MEK-inhibitor versus BRAF-inhibitor*																										
Ascierto et al. 2016, A, AU, B, CDN, CH, D, E, F, GB, H, I, ISR, NL, NO, NZ, R, S, US[coBRIM]	[1] COB (60 mg 1x daily for 3 weeks, followed from day 1 to day 21 in each 28-day cycle) + VEM (960 mg 2x daily)	[2] VEM (960 mg 2x daily) + placebo	*N*= 495 [1] 247 [2] 248	1:1	[1] 56 [2] 55	42.2	225742519050000DB	14.2	[1] 12.3 [2] 7.2HR: 0.58 [95% CI: 0.46–0.72; *p*<0.0001]	n.s.	n.s.	n.s.	n.s.	n.s.	n.s.	[1] 22.3 [2] 17.4HR: 0.70 [95% CI: 0.55–0.90; *p*=0.005]	[1] 74.5 [2] 63.8	[1] 48.3 [2] 38.0	n.s.	n.s.	n.s.	n.s.	[1] 70 [2] 50	[1] 60 [2] 52	Hoffmann-La Roche	COB plus VEM is more effective than VEM in patients with stage III or IV BRAF-mutated melanoma
Ascierto et al. 2021, A, AU, B, CDN, CH, D, E, F, GB, H, I, ISR, NL, NO, NZ, R, S, US[coBRIM]	[1] COB + VEM	[2] VEM + placebo	*N*= 495 [1] 247 [2] 248	1:1	[1] 56 [2] 55	42.2	225742520955000DB	[1] 21.2 [2] 16.6	[1] 12.6 [2] 7.2	n.s.	[1] 32 [2] 16	[1] 23 [2] 13	[1] 17 [2] 12	[1] 14 [2] 10	n.s.	[1] 22.5 [2] 17.4	n.s.	[1] 49 [2] 39	[1] 38 [2] 31	[1] 34 [2] 29	[1] 31 [2] 26	n.s.	[1] 70[2] 50	[1] 78 [2] 63	Hoffmann-La Roche	The 5-year analysis shows improved effectiveness with COB plus VEM versus VEM in patients with BRAF-mutated stage III or IV melanoma.
Dummer et al. 2018, AMS, ARG, AU, BRA, CDN, CH, COL, COR, D, E, F, GB, GR, H, I, ISR, J, MEX, NL, NO, P, PL, R, S, SIN, SLK, TRK, US[COLUMBUS]	[1] ENCO (450 mg 1x daily) + BIN (45 mg 2x daily)	[2] VEM (960 mg 2x daily)[3] ENCO (300 mg 2x daily)	*N*= 577 [1] 192 [2] 191 [3] 194	1:1:1	55	42.1	O	742950383857500 [1] 16.7 [2] 14.4 [3] 16.6	[1] 14.9 [2] 7.3 [3] 9.6 [1] vs. [2]: HR: 0.54 [95% CI: 0.41–0.71; *p*<0.0001] [1] vs. [3]: HR: 0.75 [95% CI: 0.56–1.00; *p*=0.051]	n.s.	n.s.	n.s.	n.s.	n.s.	n.s.	n.s.	n.s.	n.s.	n.s.	n.s.	n.s.	n.s.	[1] 63 [2] 40 [3] 51	[1] 58 [2] 63 [3] 66	Array BioPharma und Novartis Pharmaceuticals	ENCO plus BIN is more effective than VEM and ENCO in patients with stage III or IV BRAF-mutated melanoma
Long et al. 2015, ARG, AU, CDN, D, E, F, GB, GR, I, NL, R, S, UKR, US[COMBI-d]	[1] DAB (150 mg 2x daily ) + TRAM (2 mg 1x daily)	[2] DAB (150 mg 2x daily) + placebo	*N*= 423 [1] 211 [2] 212	1:1	[1] 55 [2] 56.5	46.8	DB	[1] 20 [2] 16	[1] 11.0 [2] 8.8HR: 0.67 [95% CI: 0.53–0.84; *p*=0.0004]	n.s.	n.s.	n.s.	n.s.	n.s.	n.s.	[1] 25.1 [2] 18.7HR: 0.71 [95% CI: 0.55–0.92; *p*=0.0107]	[1] 74 [2] 68	[1] 51 [2] 42	n.s.	n.s.	n.s.	n.s.	[1] 69 [2] 53	[1] 32 [2] 31	GlaxoSmithKline	DAB plus TRAM is more effective in patients with BRAF-mutated stage IIIC or IV melanoma compared with DAB.
Long et al. 2017, ARG, AU, CDN, D, E, F, GB, GR, I, NL, R, S, UKR, US[COMBI-d]	[1] DAB + TRAM	[2] DAB + placebo	*N*= 423 [1] 211 [2] 212	1:1	[1] 55 [2] 56.5	46.8	DB	36	n.s.	n.s.	[1] 30 [2] 16	[1] 22 [2] 12	n.s.	n.s.	n.s.	[1] 25.1 [2] 18.7HR: 0.75 [95% CI: 0.58–0.96]	n.s.	[1] 52 [2] 43	[1] 44 [2] 32	n.s.	n.s.	n.s.	[1] 68 [2] 55	[1] 48 [2] 50	Novartis	The 3-year analysis demonstrates improved efficacy with DAB plus TRAM versus DAB in patients with BRAF-mutated stage IIIC or IV melanoma.
Robert et al. 2015, A, ARG, AU, B, BRA, CDN, COR, D, DK, E, F, FIN, GB, H, I, IR, ISR, NL, NO, NZ, PL, R, S, TWN, UKR, US[COMBI-v]	[1] DAB (150 mg 2x daily) + TRAM (2 mg 1x daily)	[2] VEM (960 mg 2x daily)	*N*= 704 [1] 352 [2] 352	1:1	55	45	O	11	[1] 11.4 [2] 7.3HR: 0.56 [95% CI: 0.46–0.69; *p*<0.001]	n.s.	n.s.	n.s.	n.s.	n.s.	n.s.	[1] >12 [2] 17.2HR: 0.69 [95% CI: 0.53–0.89; *p*=0.005]	[1] 72 [2] 65	n.s.	n.s.	n.s.	n.s.	n.s.	[1] 64 [2] 51	[1] 52 [2] 63	GlaxoSmithKline	DAB plus TRAM is more effective than VEM in patients with BRAF-mutated stage IIIC or IV melanoma
*PD-1-inhibitor plus MEK-inhibitor versus PD-1-inhibitor*																										
Gogas et al. 2020a, AU, B, BRA, COR, D, E, F, GB, GR, H, I, NL, PL, R, US[IMspire170]	[1] COB (60 mg 1x daily) + ATE (840 mg day 1 und 15 in each 28-day cycle)	[2] PEM (200 mg every 3 weeks)	*N*= 446 [1] 222 [2] 224	1:1	66	39.5	O	[1] 7.1 [2] 7.2	[1] 5.5 [2] 5.7HR: 1.15 [95% CI: 0.88–1.50; *p*=0.30]	[1] 30 [2] 39	n.s.	n.s.	n.s.	n.s.	n.s.	[1] >12 [2] >12HR: 1.06 [95% CI: 0.69–1.61]	n.s.	n.s.	n.s.	n.s.	n.s.	n.s.	[1] 26 [2] 31.6	[1] 66.8 [2] 33.3	Hoffmann-La Roche	COB plus ATE is not more effective than PEM in patients with stage III or IV BRAF wild-type
*PD-1-inhibitor plus IDO-inhibitor versus PD-1-inhibitor*																										
Long et al. 2019a, AMS, AU, B, CDN, CH, COR, D, DK, E, F, GB, I, IR, ISR, J, MEX, NO, NZ, PL, R, S, US[ECHO-301]	[1] PEM (200 mg every 3 weeks) plus EPA (100 mg 2x daily)	[2] PEM (200 mg every 3 weeks) + placebo	*N*= 706 [1] 354 [2] 352	1:1	[1] 64 [2] 63	40.1	DB	12.4	[1] 4.7 [2] 4.9HR: 1.00 [95% CI: 0.83–1.21; *p*=0.52]	[1] 36.9 [2] 36.6	n.s.	n.s.	n.s.	n.s.	n.s.	[1] >12 [2] >12HR: 1.13 [95% CI: 0.86–1.49; *p*=0.81]	[1] 74.4 [2] 74.1	n.s.	n.s.	n.s.	n.s.	n.s.	[1] 34 [2] 32	[1] 22 [2] 17	Incyte Corporation	EPA plus PEM is not more effective compared with PEM in stage III or IV patients

*A* Austria, *AMS* South America, *ARG* Argentina, *ATE* atezolizumab, *AU* Australia, *B* Belgium, *BIN* binimetinib, *BRA* Brazil, *BRAF* B-rapidly accelerated fibrosarcoma protein, *CDN* Canada, *CH* Switzerland, *COB* cobimetinib, *COL* Colombia, *COR* Korea, *CS* Czech Republic, *CTLA-4* cytotoxic T-lymphocyte-associated protein 4, *D* Germany, *DAB* dabrafenib, *DB* double-blind, *DK* Denmark, *E* Spain, *ENCO* encorafenib, *EPA* epacadostat, *F* France, *FIN* Finland, *GB* United Kingdom, *GR* Greece, *H* Hungary, *HR* hazard ratio, *I* Italy, *IDO* indoleamine 2,3-dioxygenase, *IPI* ipilimumab, *IR* Ireland, *ISR* Israel, *J* Japan, *KI* confidence interval, *MEK* mitogen-activated protein kinase, *MEX* Mexico, *N* study size, *NIVO* Nivolumab, *NL* Netherlands, *NO* Norway, *NZ* New Zealand, *n.s.* not specified, *O* Open, *OS* overall survival, *P* Portugal, *PD-1* programmed cell death protein 1, *PEM* pembrolizumab, *PFS* progression-free survival, *PL* Poland, *R* Russia, *S* Sweden, *SIN* Singapore, *SLK* Slovakia, *TRAE* treatment-related adverse events, *TRAM* trametinib, *TRK* Turkey, *TWN* Taiwan, *UKR* Ukraine, *US* United States of America, *VEM* vemurafenib

^*^The study design is not designed for a comparison between the combination drug and nivolumab. The analysis is performed without formal hypothesis testing. Therefore, only descriptive *p*-values are reported

### Effectiveness of combination therapy compared to monotherapy

Eleven studies investigated the effectiveness of combination therapy versus monotherapy in stage III or IV MM. In six studies patients were treated with targeted therapies [13, 14, 17–20] and in four studies patients treated with immunotherapies [21–24]. Only Gogas et al. investigated the combination of a targeted therapy drug with a drug of immunotherapy [25].

#### Progression-free survival

CheckMate 067 examined NIVO plus IPI compared with NIVO and IPI with a follow-up of at least 77 months. Results related to PFS were significant at all time points in favor of NIVO plus IPI versus IPI (hazard ratio (HR): 0.42 [95% CI: 0.35–0.51; *p* < 0.0001]). The risk of disease progression (DP) was reduced by 58% with NIVO plus IPI compared to IPI [21–23]. At 48 months, more patients were progression-free with NIVO plus IPI (37%) than with NIVO (31%) and IPI (9%) [21]. While the median PFS in patients with BRAF-mutated melanoma was 16.8 months with NIVO plus IPI, 5.6 months with NIVO, and 3.4 months with IPI, median PFS in patients with BRAF wild-type was 11.2 months, 8.2 months, and 2.8 months, respectively. Overall, the results were in favor of the combination therapy [21–23]. Elevated LDH levels had an unfavorable impact on PFS. While 41% of patients with normal LDH levels were progression-free at 5 years with NIVO plus IPI, this was 28% for patients with elevated LDH levels. Patients treated with monotherapy were less likely progression-free, regardless the LDH level [22].

DAB plus TRAM was superior to DAB in COMBI-d [14, 20] and VEM in COMBI-v [13] in patients with BRAF mutation melanoma. The risk of progression was reduced by 33% with combination therapy compared to DAB (HR: 0.67 [95% CI: 0.53–0.84; *p* = 0.0004]). At 36 months, more patients were progression-free with combination therapy (22%) than with DAB (12%). While 27% of patients with normal LDH levels treated with combination therapy were still alive after 3 years, 13% of patients with elevated LDH levels were [14, 20]. Combination therapy also proved superiority to VEM. The risk of progression was reduced by 44% (HR: 0.56 [95% CI: 0.46–0.69; *p* < 0.001]) [13].

At 14.2 months of follow-up, there was an improvement in PFS with COB plus VEM compared to VEM (coBRIM). The risk of progression was significantly reduced by 42% (HR: 0.58 [95% CI: 0.46–0.72; *p* < 0.0001]) [17]. After 60 months, more patients were progression-free with combination therapy (14%) than with VEM (10%). A subgroup analysis showed that after 5 years, more patients with normal LDH levels in combination therapy (18%) were progression-free than patients with elevated LDH levels (7%) [18].

COB plus atezolizumab (ATE) resulted in a nonsignificant worsening of PFS compared to PEM (HR: 1.15 [95% CI: 0.88–1.50; *p* = 0.30]). The risk of progression increased by 15%. At 1 year, fewer patients were progression-free under combination therapy (30%) than under monotherapy (39%) [25] (IMspire170).

At a median follow-up of 12.4 months, epacadostat (EPA) plus PEM showed no significant difference in PFS compared to PEM (HR: 1.00 [95% CI: 0.83–1.21]; *p* = 0.52). At one year, as many patients with combination therapy (37%) were progression-free as with PEM (37%). The lack of benefit in terms of PFS with additional administration of EPA was also evident in subgroup analysis by baseline BRAF status and LDH level [24] (ECHO-301).

With a median follow-up of 16.6 months the therapy with ENCO plus BIN resulted in a significant 46% reduction in risk of progression compared to VEM (HR: 0.54 [95% CI: 0.41–0.71; *p* < 0.0001]). The combination therapy was also superior to ENCO with a median PFS of 9.6 months (95% CI: 7.5–14.8). Data on PFS rates were not available (COLUMBUS) [19].

#### Overall survival

NIVO plus IPI improved OS compared to NIVO and IPI in CheckMate 067. The combination therapy achieved a median follow-up of 72.1 months (95% CI: 38.2—not reached). This was lower for NIVO (36.9 months [95% CI: 28.2–58.7]) and IPI (19.9 months [95% CI: 16.8–24.6]). These results confirm the long-lasting survival benefit with NIVO plus IPI. However, OS rates in all study groups declined over time. After 78 months, more patients were alive with NIVO plus IPI (49%) than with IPI (23%) and NIVO (42%) [23]. The combination therapy resulted in a significant 48% increase in OS compared to IPI (HR: 0.52 [95% CI: 0.42–0.64; *p* < 0.0001]) and a 16% reduction in risk of death (HR: 0.84 [95% CI: 0.67–1.05]) compared to NIVO [21–23].

OS was longer with NIVO plus IPI than with NIVO and IPI in the subgroups of patients with BRAF mutation and wild-type. At 6.5 years, more patients with BRAF mutation were alive on the combination therapy (57%) than on NIVO (43%) and IPI (25%). Compared to patients with BRAF wild-type, fewer patients with BRAF wild-type were alive (NIVO plus IPI: 46%, NIVO: 42%, and IPI: 25%) [23]. Elevated LDH level has an unfavorable impact on OS. The 5-year OS rate in patients with normal LDH levels was 60% with combination therapy, 53% with NIVO, and 34% with IPI. In patients with elevated LDH levels, these were 38%, 28%, and 15%, respectively [22] (CheckMate 067).

DAB plus TRAM improved OS compared to VEM in patients with BRAF mutation with a median follow-up of 11 months in COMBI-v. At 12 months, 72% of patients with combination therapy and 65% of patients with VEM were still alive. The combination therapy significantly reduced the risk of death by 31% (HR: 0.69 [95% CI: 0.53–0.89; *p* = 0.0005]) [13].

In addition to VEM, DAB plus TRAM also proved superiority to DAB in COMBI-d. DAB plus TRAM resulted in a significant 25% reduction in risk of death (HR: 0.75 [95% CI: 0.58–0.96; *p* = 0.0107]). Over 36 months, more patients were alive with NIVO plus IPI (44%) than with DAB (32%). While 54% of patients with normal LDH levels treated with the combination therapy were still alive after three years, only 25% in patients with elevated LDH levels were [14, 20].

COB plus VEM improved OS over VEM. The combination therapy significantly reduced the risk of death by 30% (HR: 0.70 [95% CI: 0.55–0.90; *p* = 0.005]) in coBRIM [17, 18]. At five years, outcomes remained in favor of combination therapy (31%) compared to monotherapy (26%). In addition, Ascierto et al. showed that patients with normal LDH levels treated with combination therapy had longer survival than patients with elevated LDH levels (43% vs. 16%) [18].

EPA plus PEM was not more effective in terms of OS compared to PEM. The combination therapy resulted in a 13% increased risk of death. However, the result was not significant (HR: 1.13 [95% CI: 0.86–1.49; *p* = 0.81]). OS at one-year was approximately 74% in both treatment groups. In addition, subgroup analysis was performed according to BRAF status and baseline LDH levels. In all subgroups, the combination therapy did not result in a significant overall survival benefit compared to monotherapy (ECHO-301) [24].

At a median follow-up of approximately 7 months, the combination of the PD-1 inhibitor COB and the MEK inhibitor ATE did not achieve median OS. The combination therapy resulted in a 6% increased risk of death compared to PEM. However, the result is not significant (HR: 1.06 [95% CI: 0.69–1.61) (IMspire170) [25].

#### Objective response rate

NIVO plus IPI (58%) showed improved ORR compared to NIVO (45%) and IPI (19%) [23]. Over 6.5 years, the values were stable and favored the combination therapy (CheckMate 067) [21–23]. The median duration of response was not reached with the combination therapy and NIVO. With IPI, it was 19.2 months. In addition, Wolchok et al. found a correlation between ORR and PFS and OS. Thus, patients with combination therapy with objective response within the first 12 months showed high sustained PFS and OS rates compared to patients with NIVO and IPI. The proportion of patients requiring post-trial treatment was lower with combination therapy (36%) than with NIVO (49%) and IPI (66%) [23].

The ORR was significantly higher with DAB plus TRAM (64% [95% CI: 59.1–69.4; *p* < 0.001]) than with VEM (51% [95% CI: 46.1–56.8; *p* < 0.001]). The median duration of response with VEM was (7.5 months [95% CI: 7.3–9.3]) 4 months shorter than with combination therapy (13.8 months [95% CI: 11.0-not reached]) (COMBI-v) [13].

Like COMBI-v, COMBI-d also showed significantly higher ORR with DAB plus TRAM but compared to DAB rather than VEM. COMBI-d showed a significant difference in ORR of 15% (95% CI: 6–25; *p* = 0.0014) in favor of the combination therapy (69% [95% CI: 61.5–74.5])) versus DAB (53% [95% CI: 47.8–61.5]) [14]. The median duration of response was slightly longer with combination therapy (12.0 months [95% CI: 9.3–17.1]) than with DAB (10.6 months [95% CI: 8.3–12.9]) [14, 20].

In coBRIM, there was a 20% significantly higher ORR with COB plus VEM (70% [95% CI: 63.5–75.3; *p* < 0.0001]) than with VEM (50% [95% CI: 43.6–56.4; *p* < 0.0001]). In the primary analysis by Ascierto et al., the median duration of response was longer with combination therapy (13.0 months [95% CI: 11.1–16.6]) than with VEM (9.2% [95% CI: 7.5–12.8]) [17]. In the 5-year analysis, Ascierto et al. noted an improvement from 13.0 to 14.7 months (95% CI: 12.9–19.3) with the combination therapy [18].

IMspire170 observed an ORR that was 6% lower with COB plus ATE (26% [95% CI: 20.1–32.6]) than with PEM (31.6% [95% CI: 25.3–38.4]). Combination treatment non-significantly reduces the chance of objective response by 23% (OR: 0.77 [95% CI: 0.5–1.18]) [25].

Results from COLUMBUS showed an improvement in response with ENCO plus BIN compared with ENCO and VEM. Patients achieved a 63% response with ENCO plus BIN (95% CI: 55.8–69.9), whereas only 51% with ENCO (95% CI: 43.3–57.8) and 40% with VEM (95% CI: 33.3–47.6). The median duration of response was longer with the combination therapy (16.6 months [95% CI: 12.2–20.4]) than with ENCO (14.9 months [95% CI: 11.1-not assessable]) and VEM (12.3 months [95% CI: 6.9–16.9]) [19].

In ECHO-301, the addition of EPA to PEM did not improve ORR compared to PEM. In both groups, ORR was similar at approximately 30%. With a median follow-up of 12.4 months, neither the patients on the combination therapy nor the patients on the monotherapy achieved the median duration of response [24].

#### Adverse events

Studies have found that combination therapies are predominantly more effective than monotherapies in terms of survival and tumor response. Although adverse events are a measure of safety in drug use, an impact on effectiveness cannot be ruled out.

In CheckMate 067, Grade 3 or 4 treatment-related adverse events (TRAE) occurred in 59% of patients with NIVO plus IPI. These were more than twice as common in patients than with NIVO (23%) and IPI (28%) [21–23]. The study reported Grade 3 or 4 TRAE occurred with a frequency of ≥ 5% in each group [21–23]. Approximately one-third of patients on combination therapy discontinued therapy prematurely due to TRAE. Treatment discontinuation rates with NIVO (8%) and IPI (14%) were significantly lower [23]. A total of four patients died. Of these, two deaths were attributable to NIVO plus IPI and one each to NIVO and IPI [21–23]. There was no dose reduction or interruption. OS and PFS rates in patients who discontinued treatment with combination therapy due to TRAE were comparable to survival rates in the overall population. Therefore, an unfavorable impact of treatment discontinuation on effectiveness was excluded [22].

While the incidence of TRAE was more than double in immunotherapy with NIVO plus IPI compared to monotherapy, there was no significant difference between patients receiving targeted therapy with DAB plus TRAM (52%) or VEM (63%). Robert et al. reported grade 3 or 4 TRAE occurring at a frequency of ≥ 10% in each study group. The rate of treatment discontinuation was similar for DAB plus TRAM (13%) and VEM (12%). No treatment-related deaths occurred. The authors reported a similarly high rate of grade 3 or 4 TRAEs leading to dose reduction or discontinuation. However, values are missing because no specification by grade was made. Overall, dose reductions occurred in 33% of patients receiving DAB plus TRAM and in 39% of patients receiving VEM. In both groups, the frequency of dose interruption was similar at approximately 55% (COMBI-v) [13].

Long et al. reported grade 3 or 4 TRAE occurring in ≥ 10% of patients in each study group. In COMBI-d, the incidence of TRAE with DAB plus TRAM was same as in COMBI-v. The incidence with DAB (50%) was like that with combination therapy (48%) [20]. There was only one confirmed death that was associated with DAB [14, 20]. The occurrence of TRAE led to dose interruption in more than half (58%) of patients treated with combination therapy. However, this is not specified by grade [20]. 11% of patients on DAB plus TRAM and 7% on DAB permanently discontinued treatment [14]. With an additional 13 months of follow-up, this rate increased by 3% with combination therapy [20].

In coBRIM, Grade 3 or higher TRAE occurred in ≥ 2% of patients in each study group. At a median follow-up of 21.2 months, more TRAEs occurred with COB plus VEM (78%) than with VEM (63%) [18]. The occurrence of TRAE increased over time [17]. During the study, the rate of treatment discontinuation increased from 14 to 27% with combination therapy and from 7 to 12% with VEM. The study did not distinguish between the grades of TRAE that led to treatment discontinuation [18]. Dose reductions occurred in patients with combination therapy. Physicians reduced the dose of VEM in 35% and of COB in 30% of patients. In comparison, the rate of dose reduction was slightly lower for monotherapy (29%) [17]. Overall, six deaths were attributable to the combination therapy and five to the monotherapy [18].

IMspire170 reported grade ≥ 3 TRAE occurring in ≥ 2% of patients in each study group. The incidence of TRAE was twice as high with COB plus ATE (67%) than with PEM (33%). TRAE resulted in dose reduction or discontinuation in 72% of patients with combination therapy and in 27% of patients with PEM. No distinction was made between the grades of TRAE. The study showed no significant association between COB dose intensity and PFS (HR: 1.31 [95% CI: 0.91–1.88]), ATE dose intensity and PFS (HR: 1.52 [95% CI: 1.04–2.21]), and COB dose reduction and PFS (HR: 1.26 [95% CI: 0.86–1.85]). Among patients treated with the combination therapy, one agent (21%) or both agents (12%) were permanently discontinued. For monotherapy, the discontinuation rate was 6%. Three deaths occurred due the combination therapy and two due the monotherapy [25].

Dummer et al. reported grade ≥ 3 TRAE occurring in ≥ 2% of patients in each study group. TRAE occurred less frequently with ENCO plus BIN (58%) than with ENCO (66%) and VEM (63%). Patients receiving the combination therapy discontinued therapy less frequently (13%) than patients treated with ENCO (14%) or VEM (17%). The combination therapy resulted in fewer dose interruptions (46%) or adjustments (11%) than ENCO (64% and 27%, respectively) and VEM (53% and 23%, respectively). No difference was made between grades of AEs that resulted in treatment discontinuation or dose adjustment. No treatment-related deaths occurred (COLUMBUS) [19].

Grade ≥ 3 TRAE occurred more frequently in patients treated with EPA plus PEM (22%) than in patients treated with PEM (17%) [24]. TRAE led to dose interruption in combination therapy (22%) and in monotherapy (19%). In both study groups, 10% of patients discontinued therapy prematurely. Dose reduction occurred in 8% of patients in each group. For combination therapy, only one dose reduction of EPA occurred. No treatment-related deaths occurred [24].

Predominantly more TRAE lead to treatment discontinuation, dose reduction, or interruption with combination therapies than monotherapies. The impact on effectiveness remains mostly unclear. Nevertheless, combination therapy was more effective than with monotherapy in 9 of 11 trials.

### Study characteristics of the cost–utility analyses

Table 2 provides an overview of all included cost–utility analyses [26–30]. One study considered only stage III patients [26]. While one study only considered patients with BRAF mutation [27], studies examining PD-1 and CTLA-4 inhibitors included patients with BRAF wild-type and mutation [26, 28–30]. Three studies received financial support from the pharmaceutical industry [26, 28, 29], one was independently funded [27] and one had no funding information [30]. While two studies used a Markov modeling approach [26, 27], three studies used a partitioned survival model with three health states [28–30]. All analyses consider direct costs from payer perspective. While two studies examined a lifelong time horizon, two studies used time horizons of 20 and 30 years. All studies used the same discount rate for costs and outcomes that varied between 2 and 6%.

**Table 2:** Overview of the included cost–utility analyses

Authors, year, country	Intervention	Comparator	Model	Perspective, cost type, time horizon	Discount rate per year [%]	Costs	QALY	ICER	Max. WTP	Sponsor	Conclusion
*BRAF-inhibitor plus MEK-inhibitor versus PD-1-inhibitor or BRAF-inhibitor*											
Bensimon et al., 2020, US	[1] DABmax12 (150 mg 2 × daily) + TRAMmax12 (2 mg 1 × daily)	[2] PEMmax12 (200 mg every 3 weeks)	Markov model with four states (recurrence-free, loco-regional recurrence, distant metastases, and death)	Payer, direct costs, lifelong	Costs: 3 Effectiveness: 3	Base case: [1] 583.588 USD [2] 520.812 USD	Base case: [1] 8.15 [2] 9.07	[2] is dominant	100.000 USD	Merck Sharp & Dohme	PEM is dominant over DAB plus TRAM with lower costs and higher QALYs
Matter-Walstra et al., 2015, Switzerland	[1] DAB (150 mg 2 × daily) + TRAM (2 mg 1 × daily)	[2] VEM (960 mg 2 × daily)	Markov model with three health states (progression-free, disease progression, and death)	Payer, direct costs, lifelong	Costs: 3 and 6 Effectiveness: 3 and 6	Base case: [1]: 311.421 CHF [2]: 111.773 CHF 3% discount rate: [1]: 302.747 CHF [2]: 110.187 CHF 6% discount rate: [1]: 294.984 CHF [2]: 108.730 CHF	Base case: [1]: 1.54 [2]: 1.02 3% discount rate: [1]: 1.49 [2]: 1.0 6% discount rate: [1]: 1.45 [2]: 0.99	Base case: 385.603 CHF/QALY 3% discount rate: 395.204 CHF/QALY 6% discount rate: 404.542 CHF/QALY	100.000 CHF	State secretariat for Education, Research, and Innovation	At the maximum WTP of CHF 100,000/QALY, DAB plus TRAM is not cost-effective compared to VEM in Switzerland
*PD-1-inhibitor plus CTLA-4-inhibitor versus CTLA-4-inhibitor or PD-1-inhibitor*											
Paly et al., 2020, Japan	[1] NIVO (1 mg/kg) + IPI (3 mg/kg) once every 3 weeks for 4 doses, then NIVO (3 mg/kg) only every 2 weeks	[2] NIVO (240 mg every 2 weeks) [3] IPI (3 mg/kg every 3 weeks for a total of 4 doses)	Partitioned three-state survival model (pre-progression, post-progression, and death)	Payer, direct costs, 30 years	Costs: 2 Effectiveness: 2	Base case: [1] 180.649,54 USD [2] 169.958,06 USD [3] 109.314,61 USD	Base case l: [1] 7.7 [2] 6.2 [3] 2.8	[1] vs. [2] 7.000 USD/QALY [1] vs. [3] 15.000 USD/QALY	69.000 USD	Bristol-Myers Squibb	NIVO plus IPI is cost-effective compared to NIVO and IPI
Quon et al., 2019, Canada	[1] NIVO (1 mg/kg) + IPI (3 mg/kg) once every 3 weeks for 4 doses, then NIVO (3 mg/kg) only every 2 weeks	[2] NIVO (3 mg/kg) once every 2 weeks + placebo [3] IPI (3 mg/kg) once every 3 weeks for a total of 4 doses + placebo [4] PEMmax24 (2 mg/kg every 3 weeks) [5] PEMmaxKP (2 mg/kg every 3 weeks)	Partitioned three-state survival model (progression-free survival, survival after progression, and death)	Payer, direct costs, 20 years	Costs: 5 Effectiveness: 5	Base case: [1] 289.085 CAND [2] 262.271 CAND [3] 139.529 CAND [4] 154.317 CAND [5] 335.634 CAND	Base case: [1] 4.05 [2] 3.48 [3] 1.81 [4] 2.47 [5] 2.47	[1] vs. [2] 47.119 CAND/QALY (36.000 USD/QALY) [1] vs. [3] 66.750 CAND/QALY (51.000 USD/QALY) [1] vs. [4] 85.436 CAND/QALY [1] vs. [5] dominant	100.000 CAND	Bristol-Myers Squibb	NIVO plus IPI is shown to be a cost-effective treatment compared with NIVO, IPI, and [4] PEM. Compared with NIVO plus IPI, [5] PEM represents a dominated strategy
Wu und Shi, 2020, US	[1] NIVO (1 mg/kg) + IPI (3 mg/kg) once every 3 weeks for 4 doses, then NIVO (3 mg/kg) only every 2 weeks	[2] PEMmax48 (200 mg every 3 weeks)	Partitioned three-state survival model (progression-free survival, survival after progression, and death)	Payer, direct costs, lifelong	Costs: 3 Effectiveness: 3	Base case: [1] 402.221 USD [2] 236.111 USD	Base case: [1] 10,031 [2] 7,368	125.593 USD/QALY	150.000 USD	n.s	Based on the maximum WTP of $150,000/QALY, NIVO plus IPI is cost-effective compared to PEM

*BRAF* B-rapidly accelerated fibrosarcoma protein, *CAND* Canadian dollar, *CHF* Swiss franc, *CTLA-4* cytotoxic T-lymphocyte-associated protein 4, *DAB* dabrafenib, *ICER* incremental cost-effectiveness ratio, *IPI* Ipilimumab, *maxKP* maximum treatment duration to disease progression, *Max12* maximum treatment duration of 12 months, *Max24* maximum treatment duration of 24 months, *Max48* maximum treatment duration of 48 months, *MEK* mitogen-activated protein kinase, *NIVO* nivolumab, *n.s.* not specified, *PD-1* programmed cell death protein 1, *PEM* pembrolizumab, *QALY* quality-adjusted life years, *TRAM* trametinib, *USD* US dollars, *VEM* vemurafenib, *WTP* willingness to pay

In all analyses, cost estimates were based on the cost of procuring the drug, administering it, disease management, and of treating adverse events. Four studies additionally considered one-time cost of dying [26, 28–30]. Few studies considered costs of follow-up treatments due to DP [28, 30].

The determination of utility values was based on different methods. In the study by Bensimon et al., utilities for PEM were based on EQ-5D-3L questionnaire data from the KEYNOTE-054 study and a cross-sectional study using the standard Gamble method [26, 31]. In addition, Bensimon et al. used a benefit discount for TRAE of grades ≥ 3 [26]. The utilities for VEM used by Matter-Walstra et al. were based on a cross-sectional study using the standard Gamble method [27, 31]. The utilities for DAB plus TRAM were based on an EQ-5D questionnaire [32]. Paly et al. used EQ-5D data collected in CheckMate 067 and applied to the population in Japan [28, 33]. Paly et al. used an utility discount for TRAE grade 3 or 4 obtained from a cross-sectional study using the standard Gamble method [28, 31]. In the study by Quon et al., it was necessary to extrapolate OS data beyond the observed time horizon [29]. Various parametric extrapolation methods were applied. In addition to OS and PFS results, Quon et al. included best objective response rates in the modeling of treatment effects [29]. As with survival data for PEM, an indirect comparison was performed for best response rate data using the Bucher method. Utilities were calculated using the standard Gamble method. In addition, a utility discount was applied for TRAE grade 3 or 4 [29]. Utilities in the study by Wu and Shi were based on data from published literature using the standard gamble method. A benefit discount was applied to TRAE [30].

### Cost-effectiveness of combination therapy compared to monotherapy

Overall, combination therapies had higher costs than monotherapies and predominantly higher utility values than monotherapies (Table 2).

DAB plus TRAM was not cost-effective compared to VEM at a maximum WTP in Switzerland of 100,000 CHF/QALY. In the base case analysis, DAB plus TRAM resulted in an ICER of 385,603 CHF/QALY compared to VEM. Discounting costs and QALYs at 3% and 6% led to an increase in ICER to 395,204 CHF/QALY and 404,542 CHF/QALY, respectively. Sensitivity analyses showed that varying the drug prices of TRAM and DAB did not result in an ICER below the WTP. With an unchanged price of DAB and a price of TRAM close to zero, the ICER was below the WTP. In the probabilistic sensitivity analysis, the ICER was compared to possible WTPs between 50,000 and 100,000 CHF/QALY. This showed that the introduction of TRAM in Switzerland at the US market price for treatment with DAB plus TRAM is not cost-effective compared to VEM. The probability of DAB plus TRAM being cost-effective compared to VEM at existing prices was zero. With TRAM and DAB each reduced in price by 50% and a maximum WTP of 50,000 CHF/QALY, the probability of combination therapy being cost-effective compared to VEM was 3%; with a maximum WTP of 100,000 CHF/QALY 73% [27].

NIVO plus IPI resulted in an ICER of 125,593 $/QALY compared to PEMmax48. With a maximum WTP of 150,000 $/QALY the combination therapy proved to be cost-effective. The result was robust in sensitivity analysis. TRAE showed a moderate or small effect on ICER. The HR of OS had the greatest impact on ICER. In addition, a longer time horizon and lower drug prices had a favorable impact [30].

In Canada, NIVO plus IPI was cost-effective compared to pembrolizumab at a maximum treatment duration of 24 months (PEMmax24), NIVO and IPI at a maximum WTP of 100,000 CAND/QALY. NIVO plus IPI resulted in ICER of 85,436 CAND/QALY, 47,119 CAND/QALY, 66,750 CAND/QALY, respectively. While PEMmax24 was cost-effective, PEMDP was dominated by NIVO plus IPI. In sensitivity analyses, drug costs showed the greatest impact on ICER. Although the incidence of adverse events was higher with combination therapy than with monotherapy, it showed little effect on ICER. The scenario examining the cost of follow-up treatment after DP showed an improvement in the cost-effectiveness of combination therapy compared to monotherapies. This is because the combination therapy improved PFS and consequently reduced the need for follow-up treatments, which are associated with costs [29].

In Japan, NIVO plus IPI was cost-effective compared to NIVO and IPI. The combination therapy was associated with higher costs and utilities. The ICER of NIVO plus IPI was 7,000 $/QALY versus NIVO and 15,000 $/QALY versus IPI. Thus, with a maximum WTP of 69,000 $/QALY, the combination therapy was cost-effective. The result proved robustness in sensitivity and scenario analyses. While the ICER in the scenario analysis was most sensitive to the shortest time horizon of 10 years, the ICER in the deterministic sensitivity analysis was most sensitive to changes in utility values and discount rates. Nevertheless, the ICERs remained below the maximum WTP [28].

Pembrolizumab with a maximum treatment duration of 12 months (PEMmax12) dominated DAB plus TRAM with lower costs and higher utilities [ICER = -68,235 USD/QALY] [26].

## Discussion

### Summary

The innovation of combination therapies represents a turning point in the therapeutic landscape of stage III or IV MM. A comparison of the 5-year OS rate of 52% with ICI NIVO plus IPI and 31% with the BRAF–MEK-inhibitor combination COB plus VEM with the 5-year OS rate of ten to 25% with monotherapy, which was the standard of care a decade ago, demonstrates the survival benefit of combination therapies. Despite the improved effectiveness, the incidence of TRAE is substantially higher with combination therapies.

Combination therapy with BRAF and MEK inhibitors were more effective than monotherapy with a BRAF inhibitor. To date, NIVO plus IPI has been established as immunotherapy in stage III or IV patients. Another potentially effective combination therapy with ICI is currently under investigation. The phase II/III RELATIVITY-047 trial represents the most recent combination therapy. Here, relatlimab, a checkpoint inhibitor for the LAG-3 gene, in combination with NIVO, demonstrated significantly improved PFS compared to NIVO [34].

Although combination therapies have a clinically promising survival benefit, its costs were higher than for monotherapy. Nevertheless, combination therapy is more cost-effective than monotherapy in three of five studies.

### Limitations

First, comparisons between studies do not lead to valid conclusions because they might be biased by differences in patient characteristics and interventions. Although checkpoint inhibitors do not target signal transmission at the BRAF gene, but rather the PD-1 and CTLA-4 proteins, CheckMate 067 showed in a subgroup analysis that patients with BRAF mutation had higher 5-year OS compared with patients with BRAF wild-type, regardless of treatment. Head-to-head studies are needed to make a valid statement about the more effective treatment alternative for patients with BRAF mutation.

Second, in coBRIM, COLUMBUS and, COMBI-v, VEM served as a comparator to COB and VEM, DAB and TRAM, and ENCO and BIN. Patients with VEM showed a similar incidence of TRAE and effectiveness in terms of median PFS and OR. Nevertheless, it remains unexplored which of these combinations is most effective compared to VEM, given the lack of head-to-head trials.

Third, in coBRIM, COMBI-v, and ECHO-301, dose reduction occurred. In COMBI-d, dose interruption occurred. In COLUMBUS and IMspire170, both dose reduction and dose interruption happened. While no association between dose reduction and PFS was observed in IMspire170, the effects of dose reduction and interruption on effectiveness are uncertain in the other studies. It is critical to relate the results back to the dose and volume planned at baseline.

Fourth, the strength of all included cost–benefit analyses is that the results of the basic models are robust to changes in the influencing variables. However, a limitation of the Markov model used in the analysis of the cost-effectiveness of DAB plus TRAM versus VEM is the use of the US price for TRAM. TRAM was not yet approved in Switzerland at that time and therefore had no market price. Since 2016, the combination therapy has been approved for treatment there. In future trials, the use of the national drug price is essential to confirm the accuracy of the outcome. Cost-effectiveness thresholds can only serve as a benchmark when using national drug prices of the particular country to ensure accuracy and relevance to the specific healthcare system under consideration.

Fifth, limitations of the study by Paly et al. are the uncertainty in extrapolating long-term survival data and the lack of clinical data for Japan. The survival data were modeled over a 30-year time horizon by Paly et al. The authors argued that this modeling would be equivalent to modeling with a lifetime time horizon at a disease age of 60 years. However, this assumption is inconsistent with the unfavorable effect of a short time horizon on ICERs demonstrated in sensitivity and scenario analyses. It remains uncertain to what extent the rationale justifies this assumption and represents a realistic reflection of practice. Finally, another limitation is that CheckMate 067 did not include patients from Japan. Therefore, the applicability of clinical data used in the model to the Japanese population is low.

Sixth, Quon et al. demonstrated the cost-effectiveness of NIVO plus IPI versus IPI and NIVO like Paly et al., but from the Canadian payer perspective. Despite older results and a shorter time horizon, both models conclude that the combination therapy is cost-effective. However, the model by Quon et al. has the limitation that the investigators estimated PFS and OS for PEM (2 mg/kg) by indirectly comparing the effectiveness of PEM at a dose of 2 mg/kg and 10 mg/kg from the phase II KEYNOTE 002 trial. In addition, median OS for PEM from the KEYNOTE-006 and CheckMate 067 trials had not been reached at analysis time. However, Quon et al. validated the extrapolation with external data to account for the uncertainty. The results were consistent with an analysis conducted in the United Kingdom that used utility data from Canada. Thus, the model results are generalizable to the Canadian population [35]. Further, Quon et al. studied PEM with two different treatment durations. Critically, the same clinical benefit was assumed for PEMDP as for PEMmax24, so they assumed that longer treatment did not lead to any additional benefit, but only to more costs. Because of higher costs, it can be concluded that treatment with PEMDP lasted longer than 24 months. However, the assumption of Quon et al. is in contradiction with the QALYs with PEMmax48 presented in the study of Wu and Shi.

Lastly, Wu and Shi showed a higher QALY for a longer treatment duration with PEM. In addition, clinical data from Gogas et al. and Long et al. showed that the median PFS with PEMDP was at most 5.7 months. Although this is the median value, the assumption of a treatment duration to DP of more than 24 months should be critically considered, considering the significantly higher costs compared to PEMmax24. The strength of Wu and Shi's analysis is that they used multiple clinical trials to accurately estimate survival data [22, 36, 37]. In this context, however, it should be noted that Wu and Shi assumed that patient characteristics did not differ across the included studies.

### Implications for research and practice

In future, new combination therapies with fewer TRAE will be needed to avoid treatment discontinuations, which are associated with wastage of resources. In addition, an analysis of the follow-up studies, if available, is needed to support the results regarding long-term effectiveness.

Evidence for treating BRAF wild-type melanoma is limited to therapy with NIVO plus IPI. The combinations of PD-1 and MEK inhibitors and of PD-1 and IDO-1 inhibitors that have been studied are among the most innovative ones. In contrast, several combination therapies are available for the treatment of patients with BRAF mutation. The triple combination to ATE, VEM, and COB results in significantly higher PFS compared with VEM plus COB [38]. In contrast, the triple combination with spartalizumab, DAB, and TRAM versus DAB plus TRAM shows no significant difference [39]. These innovations provide a basis for further research. In addition, there is a need for head-to-head studies to find the most effective combination therapy.

Cost–utility analyses play a critical role in reimbursement. While combination therapies predominantly lead to improved effectiveness from patients' perspective, they lead to higher costs for payers. To compensate for high costs, innovative combination therapies with higher utility are needed, which favorably influence the ICER and increase the probability of cost-effectiveness considering the maximum WTP.

## Conclusion

The extent of effectiveness of combination therapy with BRAF and MEK inhibitors varied depending on the combination therapy administered and the BRAF inhibitor compared. Overall, COB plus VEM, ENCO plus BIN, and DAB plus TRAM showed superiority in terms of PFS, OS, and ORR compared to monotherapy with VEM, DAB, or ENCO in patients with BRAF mutation. For the most recent innovations, a combination therapy with the PD-1 inhibitor COB and the MEK inhibitor ATE, and the PD-1 inhibitor PEM and the IDO1 inhibitor EPA, were not more effective than the PD-1 inhibitor PEM. In Japan, Canada, and the United States, NIVO plus IPI were cost-effective compared to NIVO, IPI, and PEM. While PEM dominated over DAB plus TRAM in the United States, the combination therapy was not cost-effective over VEM in Switzerland. There remains a need for further research on combination therapies. To date, therapy for patients with BRAF wild-type has been limited to NIVO plus IPI. To confirm the long-term effectiveness of combinations of BRAF and MEK inhibitors, analysis of follow-up studies is needed. In addition, the cost-effectiveness of BRAF and MEK combination therapies compared to monotherapy remains to be investigated.

## Data Availability

Data sharing is not applicable to this article as no datasets were generated or analyzed during the current study.
